# A Microgripper with a Post-Assembly Self-Locking Mechanism

**DOI:** 10.3390/s150820140

**Published:** 2015-08-14

**Authors:** Guangmin Yuan, Weizheng Yuan, Yongcun Hao, Xiaoyi Li, Honglong Chang

**Affiliations:** Key Laboratory of Micro/Nano Systems for Aerospace, Ministry of Education, Northwestern Polytechnical University, Xi’an 710072, China; E-Mails: yuangm@nwpu.edu.cn (G.Y.); yuanwz@nwpu.edu.cn (W.Y.); haoyongcun@126.com (Y.H.); xiaoy@nwpu.edu.cn (X.L.)

**Keywords:** microgripper, self-locking, post-assembly, grip

## Abstract

In this work, we report a new design for an electrostatically actuated microgripper with a post-assembly self-locking mechanism. The microgripper arms are driven by rotary comb actuators, enabling the microgripper to grip objects of any size from 0 to 100 μm. The post-assembly mechanism is driven by elastic deformation energy and static electricity to produce self-locking and releasing actions. The mechanism enables the microgripper arms to grip for long periods without continuously applying the external driving signal, which significantly reduces the effects and damage to the gripped objects caused by these external driving signals. The microgripper was fabricated using a Silicon-On-Insulator (SOI) wafer with a 30 μm structural layer. Test results show that this gripper achieves a displacement of 100 μm with a driving voltage of 33 V, and a metal wire with a diameter of about 1.6 mil is successfully gripped to demonstrate the feasibility of this post-assembly self-locking mechanism.

## 1. Introduction

The microgripper is one of the key tools in performing the micromanipulation of micro-objects and biological tissues. Much to the benefit of progress with Micro-Electro-Mechanical System (MEMS) technology and application requirements, plenty of microgrippers with different features are constantly emerging. Kim *et al.* first reported a surface micromachined polysilicon microgripper the performed the basic grip function [1]. A microgripper using thin-film shape memory alloys (SMAs) [2,3] and SU-8 [4,5,6] as the main functional material has also been reported. Microgrippers with micropyramids on the surface of the jaws were designed to decrease the adhesive forces between the jaws and micro-objects [7] to make the gripper more practical. Volland *et al*. converted the linear motion of the microactuator into a rotational motion with a system of elastic spring beams [8]. The force sense of a microgripper is established by equipping it with semiconductor strain-gauges, as seen in [9]. Millet *et al.* presented a microgripper with an amplification mechanism to obtain a large displacement [10]. Beyeler *et al.* integrated a lateral straight comb as a force sensor to measure the grasp force [11]. Chen *et al.* solved the releasing problem using a vibrating or integrating vacuum tool [12,13]. However, the actuating mechanism of all these microgrippers is based on electrostatic [1,8,10,11,12,13], thermal [2,3,4,5,6] or piezoelectric [7,9] effects and they need a continuous power supply during the process of gripping micro objects, which may affect or even damage the micro-objects in long-lasting manipulation experiments.

In this paper, we report a novel design for an electrostatic actuated microgripper with a post-assembly self-locking mechanism based on our previous work [14,15]. Compared with existing microgrippers, the presented gripper can grip objects of any size from 0 to 100 μm and only needs a driving signal during the process of gripping and releasing micro-objects, which effectively avoids the influence of external driving signals on the gripped objects during long-lasting gripping experiments.

## 2. Design

The self-locking function of the microgripper was implemented through the post-assembly structure. The feasibility of post-assembly has been verified in [16,17] and had been used to reduce the distance between the combs of an accelerometer. The working principle can be explained as follows.

**Figure 1:**
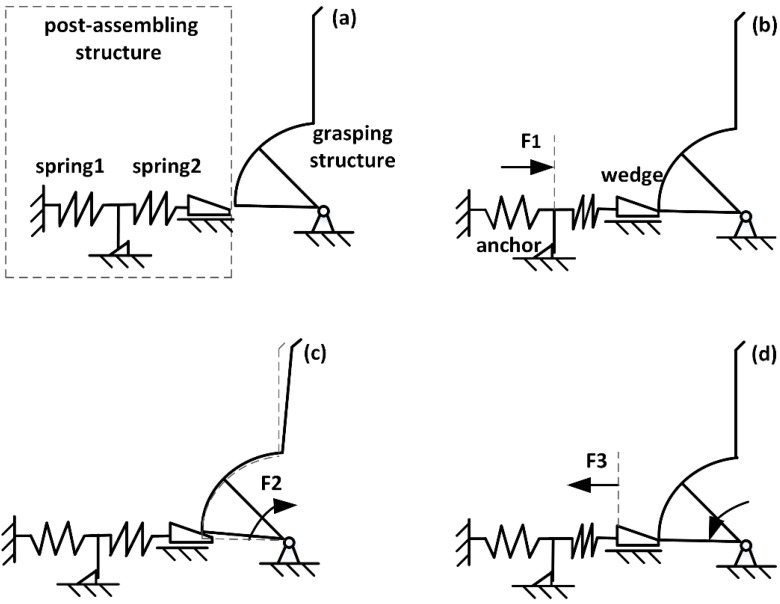
Schematic illustration of the post-assembly self-locking mechanism. (**a**) original state; (**b**) assembly; (**c**) self-locking; (**d**) releasing.

The gripper consists of two parts: the grasping structure and the post-assembly structure. Figure 1 is a schematic illustration of the post-assembly self-locking mechanism. Because the structure is symmetrical the schematic shows half. Figure 1a is the original state of the gripper. In Figure 1b, the assembling structure is pushed forward with an external force F1 until the assembling structure is occlusive with the anchor. In this stage, the wedge gets stuck in the gap under the gripper. Thus, spring2 is compressed and stores elastic energy, then the assembly is complete. In Figure 1c, the gripper is actuated by electrostatic force F2 to perform a grip action. Then, the gap under the gripper gets larger and the elastic energy stored in spring2 make the wedge move forward. When the electrostatic force F2 is removed, the gripper can keep the grip action because the wedge prevents the gripper from rotating in reverse. In Figure 1d, the wedge is pull by force F3, the gripper returns to the Figure 1b position and the grip action is released. 

**Figure 2:**
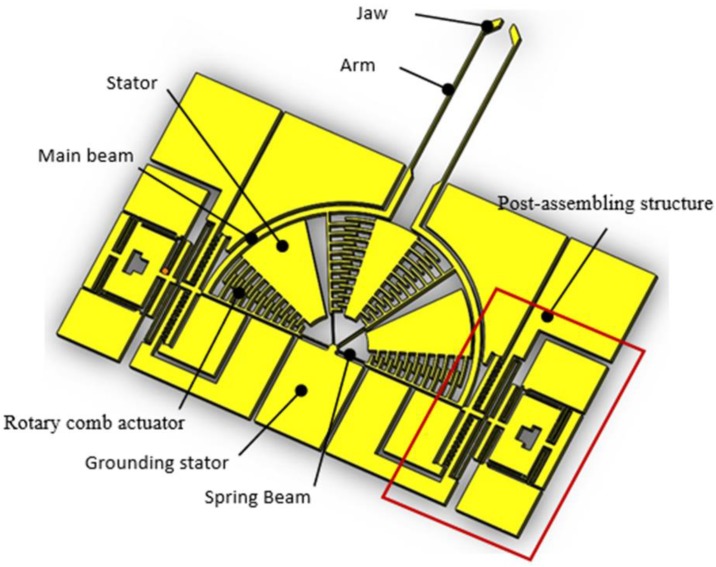
Schematic illustration of the microgripper.

The gripper presented in this paper consists of two parts: the grasping structure and the post-assembly structure, which perform grasping and self-locking functions, respectively, as shown in Figure 2. The grasping structure consists of a grounding stator, spring beam, rotary comb actuator, main beam, stator, arm and jaw. The grounding stator plays the role of supporting the moveable structures of the gripper and acts as grounding electrode. The spring beam supplies the elastic deformation. The rotary comb actuator drives the arm. The main beam serves as a rigid body to transfer displacement from actuators to the arm and jaw. The stator fixes anchor combs and supplies electrical signals. The arm is employed to magnify the displacement. The jaw contacts and grasps the micro-object directly. The microgripper’s dimensions are 4500 µm × 4000 µm × 30 µm, and it has been designed to handle objects of sizes ranging from 0 to 100 µm.

**Figure 3:**
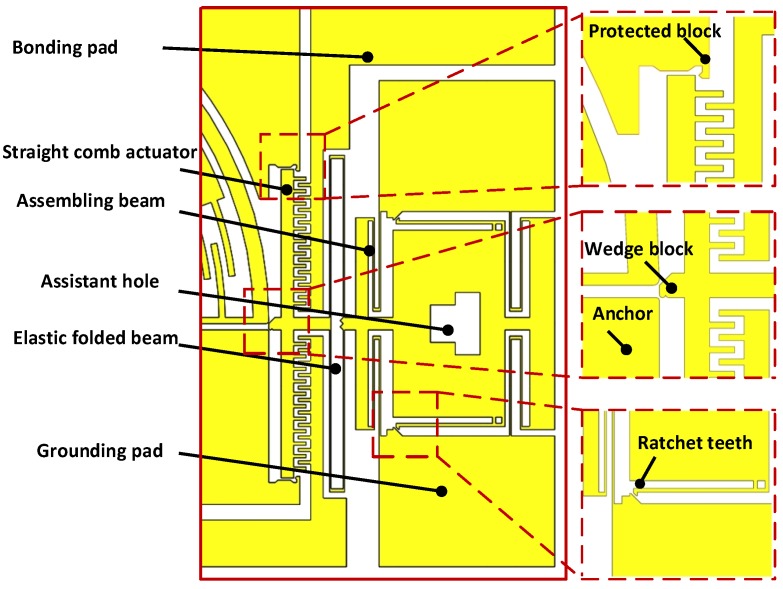
Schematic illustration of the post-assembly structure.

A detailed view of the post-assembly structure is shown in Figure 3. It consists of assistant hole, ratchet teeth, assembling beam, wedge block, straight comb actuator, bonding pad, grounding pad, elastic folded beam, anchor and protected block. Their functions can be seen through the working process of the post-assembly structure: (1) The assistant hole is pushed forward with a probe to realize the occlusion between the ratchet teeth and grounding pads, then the wedge block is automatically placed in the gap between the bottom of the main beam (as shown in Figure 2) and the anchor, forced by the elastic energy stored in the elastic folded beams; (2) The main beam is rotated by inputting an external voltage in the stator and enlarging the gap, which makes the wedge block continue to move forward. Then, the jaw holds the micro-object; (3) The external voltage is removed and self-locking is completed, because the wedge block prevents the rotation of the main beam in the opposite direction and the jaws keep holding the micro-object; (4) The preset voltage is input between the bonding pad and grounding pad,the wedge block is hauled back and the jaws are freed. Here, the role of the protected block is to prevent excessive displacement of the straight comb actuator in the lateral and hauled-back directions.

The comb actuator’s movement results from electrostatic attraction. The rotary comb actuator and straight comb actuator are driven by the driving torque and driving force, respectively. The fringe effect and the electrostatic force between the finger-tip surface and the side surface have been neglected in the theoretical analysis to simplify the comb finger model. The simplified form of the driving torque $\tau_{d}$ of the rotary comb actuator and the driving force $f_{d}$ of the straight comb actuator are given, respectively, by Equation (1) [18] and Equation (2) [19]:

(1) $$\tau_{d}=\frac{1}{2}\varepsilon_{0}h\left\{\sum_{i=1}^{n-1}{\left[\ln\frac{R_{0}+2i(W_{f}+g)}{R_{0}+2i(W_{f}+g)-g}\right]}^{-1}+\sum_{i=0}^{n-1}{\left[\ln\frac{R_{0}+(2i+1)(W_{f}+g)}{R_{0}+2i(W_{f}+g)+W_{f}}\right]}^{-1}\right\}V^{2}$$

(2) $$f_{d}=2n\frac{1}{2}\varepsilon_{0}\frac{h}{d}V^{2}$$

where $\varepsilon_{0}$ is the permittivity of the vacuum with a value of $8.85\;\times \;10^{-12}F\text{/m}$, h is the thickness of the fingers, n is the number of fingers, $R_{0}$ is the radius of the cambered first comb fingers close to the centre, $W_{f}$ is the finger width, g is the gap of the cambered fingers, d is the gap of the straight fingers, and *V* is the potential difference applied in the stator.

At the same time, both the rotary comb actuator and straight comb actuator are essentially driven by electrostatic force, the electrostatic pull-in instability [20] should be considered. We solve the pull-in problem with a mechanical method. For the straight comb actuator, the protected block is designed to solve the potential pull-in problem and the maximum displacement of the rotary comb actuator is limited by the gap in the jaws, which avoids the pull-in problem in the rotary comb actuator.

## 3. Optimization

**Figure 4:**
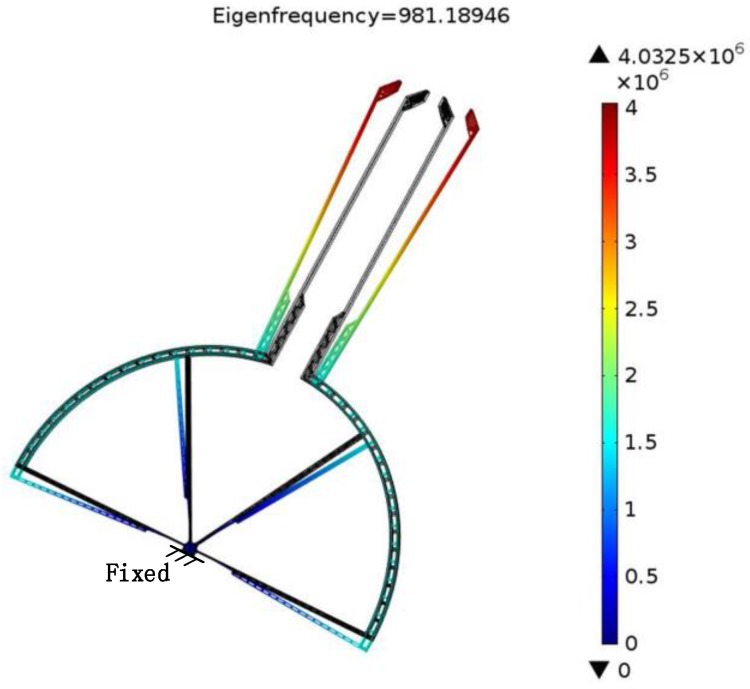
First-order natural frequency of the grasping structure.

**Figure 5:**
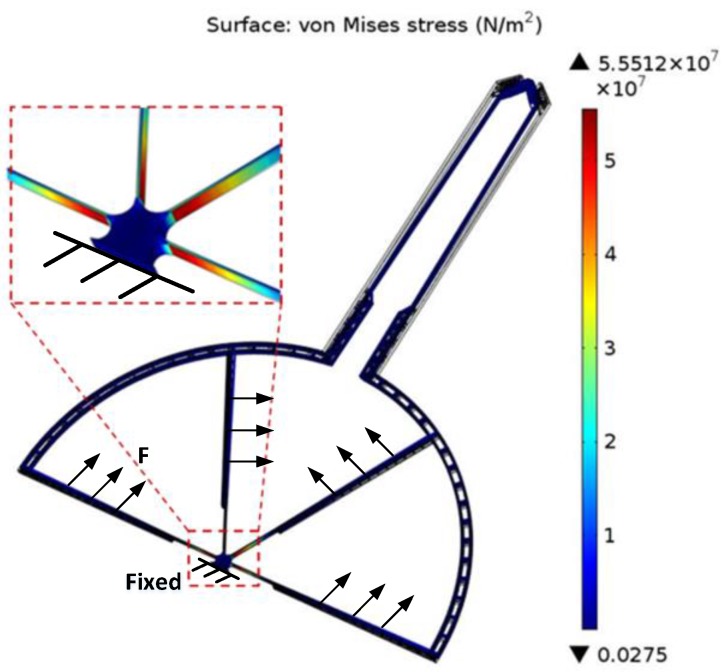
Von Mises stress contour plot of the grasping structure.

The design was optimized using the finite element analysis (FEA) software from COMSOL Multiphysics. Modal analysis and stress analysis were conducted to check the performance. In COMSOL, the materials are set to be silicon with a density of 2329 kg/m^3^, Young’s modulus of $1.7\times 10^{11}$ Pa and Poisson’s ratio of 0.22, and the mesh is genarated by automatic meshing. For all analysis, the structure is fixed at the anchor which is shown in Figure 4 and Figure 5.

### 3.1. Modal Analysis of Grasping Structure

Considering the significant influence of the frequency and vibration mode on the microgripper, they were first analyzed with COMSOL Multiphysics. Table 1 shows the first third-order natural frequencies of the grasping structure. The first-order mode’s shape, as shown in Figure 4, is in the working direction and is as expected. Its frequency is much lower than the gripper’s other modal frequency and this increases the stability during manipulation.

**Table 1:** Resonant frequency of the grasping structure.

Order	1	2	3
**Frequency (Hz)**	981	2353	8501

### 3.2. Stress Analysis

During the grasping process, the stress caused by the spring and elastic beams is positively related to deformation. In the area of the anchor and abrupt points, stress concentration would normally cause local stress. Excessive stress can break the structure and result in structural failure. This means it is necessary to evaluate the stress distribution contour plot of the microgripper.

**Figure 6:**
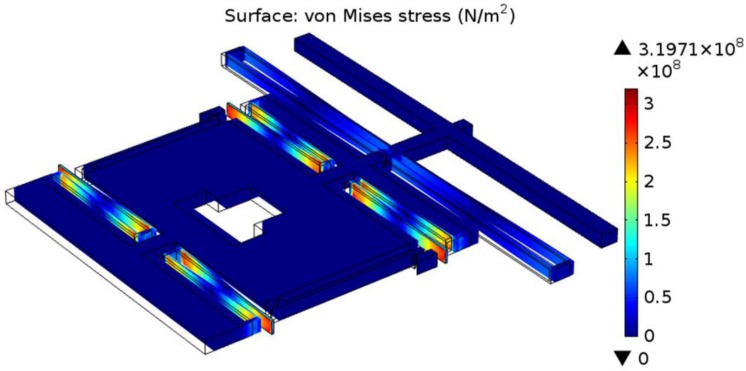
Von Mises stress contour plot of the post-assembly structure.

Figure 5 and Figure 6 show the Von Mises stress contour plot of the grasping structure and post-assembly structure. The maximum stresses of the grasping structure and post-assembly structure are about 56 MPa and 320 MPa, respectively, and occur around the anchor or the corner of the folded beam. The maximum stress of the microgripper is much less than the yield strength of single-crystal silicon, which is 7 GPa [21]. For grasping structure, the evenly distributed force with a value of 33 V is applied on the comb beams to make the jaws produce a displacement of 50 µm.

## 4. Fabrication

**Figure 7:**
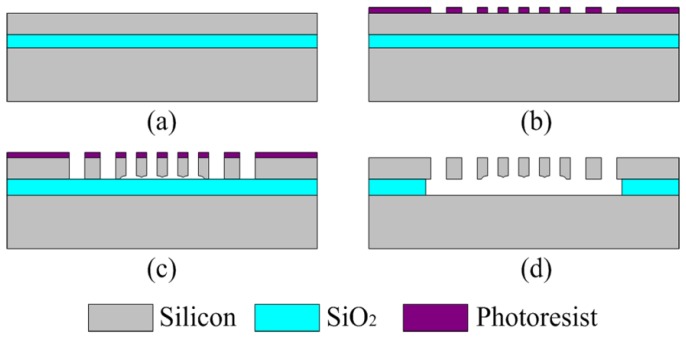
Microgripper fabrication process based on an Silicon-On-Insulator (SOI) wafer.

The microgripper was fabricated based on an Silicon-On-Insulator (SOI) wafer of which has a 400 μm thick substrate, a 4 μm thick SiO_2_ and a 30 μm thick device layer. The device layer is n-type (100) silicon with a resistivity of 0.01–0.02 Ω·cm. The fabrication process is illustrated in Figure 7.

The microgripper was fabricated using the single mask selective release process [22].The main steps are as follows: (a) Clean the SOI wafer using hydrofluoric acid solution to enhance the adhesiveness of photoresist; (b) Pattern the photoresist to the SOI wafer; (c) High aspect ratio DRIE process with several minutes over etching to deal with the problem of adhesion in the next step; (d) Remove photoresist and release the structure. The device fabrication can move freely after the release via hydrofluoric acid solution and the fabrication is accomplished.

**Figure 8:**
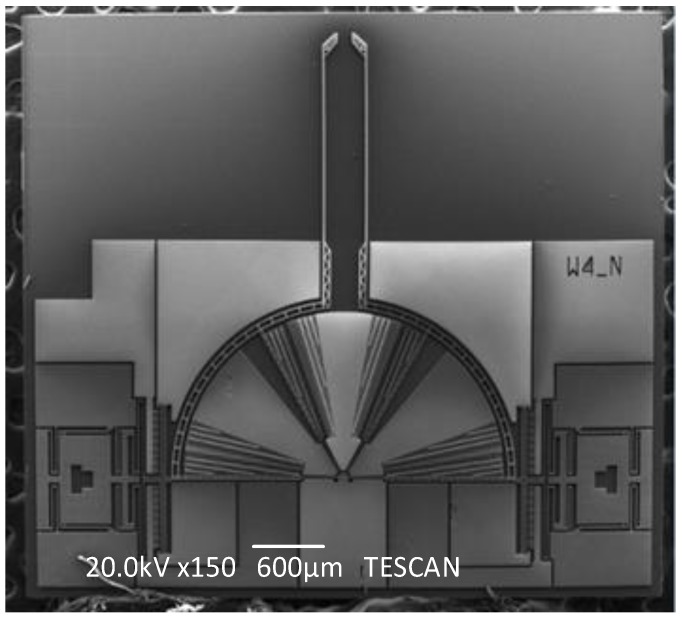
The general Scanning Electron Microscope(SEM) view of the fabricated microgripper.

**Figure 9:**
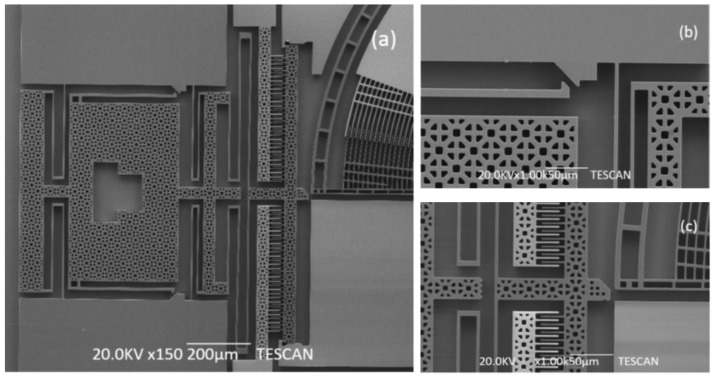
SEM pictures of post-assembly structure before assembling. (**a**) The general post-assembly structure; (**b**) the assembly structure; (**c**) the wedge block.

**Figure 10:**
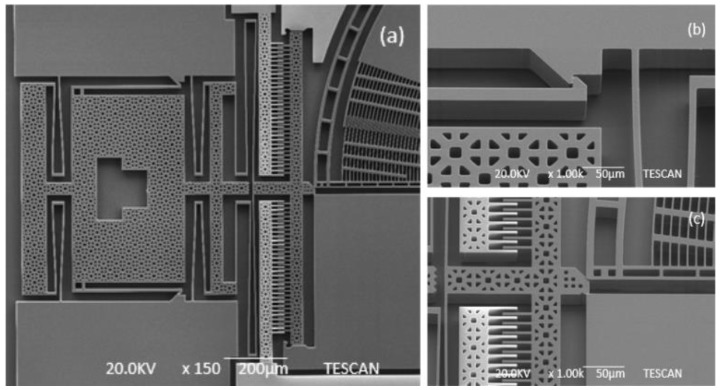
SEM pictures of post-assembly structure after assembly. (**a**) The general post-assembly structure; (**b**) the assembly structure; (**c**) the wedge block.

**Figure 11:**
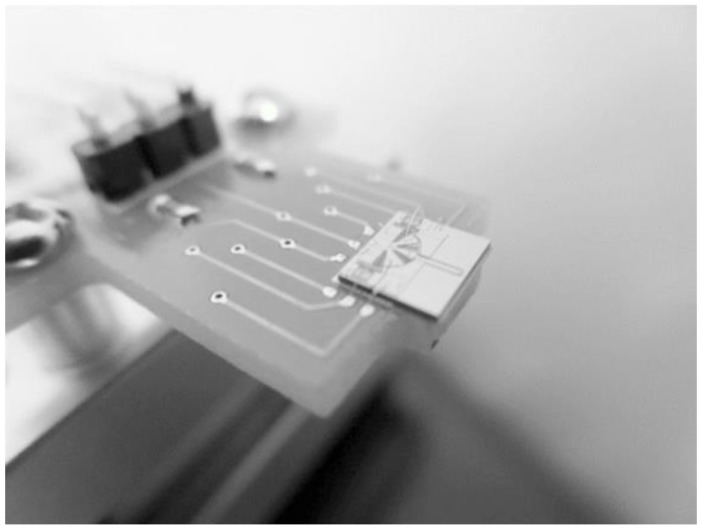
The PCB with glued and wire-bonded chip.

The general Scanning Electron Microscope (SEM) photo of the fabricated microgripper is shown in Figure 8. The overall size of the chip is 4500 μm × 4000 μm. The chip should be assembled before working. Figure 9 shows the SEM picture of the post-assembly structure before assembly and Figure 10 shows the picture after assembly, as a comparison. When assembly is finished, the gripper chip is glued and bonded directly on a printed circuit board (PCB) to access the drive signal as shown in Figure 11.

## 5. Tests and Results

**Figure 12:**
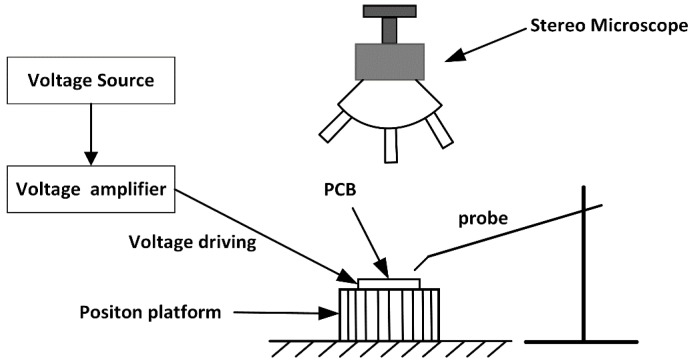
Schematic illustration of the experimental setup for the gripping test.

A peripheral control and observation system is necessary to operate the microgripper. As shown in Figure 12, the control and observing system consists of a printed circuit board (PCB) as the microgripper carrier, a position platform with three degrees of freedom (3-DOF) to control the microgripper’s position, a transport probe to inject samples, a voltage source to supply the driving voltage, a high-voltage amplifier to increase the driving signal, and a stereo microscope for observation. Here, the maximum output voltage of the high voltage amplifier is 200 V. The positioning system has three degrees of freedom (3-DOF) and has a resolution of 10 μm in all motion directions. The stereo microscope with a maximum amplification factor of 40 was selected to observe the process because of its better perfomance in terms of view depth than general microscopes. 

**Figure 13:**
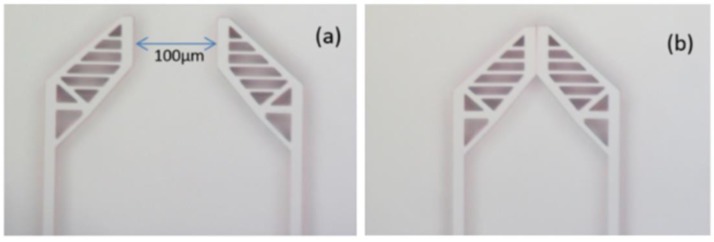
The positional relationship of the jaws when applying voltage. (**a**) Jaws open without applied voltage; (**b**) jaws closed with applied voltage of 33 V.

**Figure 14:**
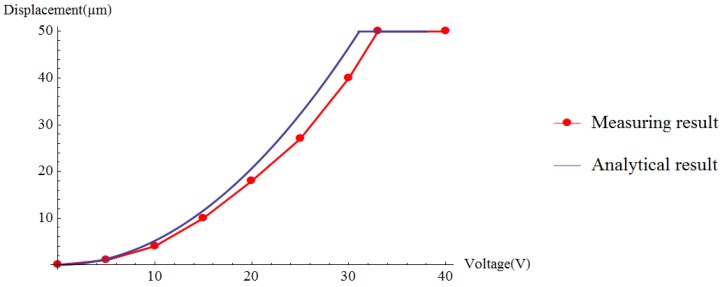
Comparison of simulation result and measuring result.

The displacement of the jaws changes with the voltage in the range of 0 V to 33 V. Figure 13 shows the distance between the jaws, which was 100 μm and 0 μm at 0 V and 33 V, respectively. Without gripped targets, the displacement is recorded as the voltage changes. As presented in Figure 14, the red dots are the measured values of the driving voltage corresponding to the different displacements. The red line is the fitting curve of the measured value of the driving voltage and the blue line is the analytical relationship between the voltage and the displacement. Table 2 shows the relationship between the displacements and voltages.The discrepancy between the analytical value and the measured results mainly comes from the approximation in the formula derivation and machining error in the MEMS fabrication. During formula derivation, we neglected the fringe effect and the electrostatic force between the finger-tip surface and the side surface for their minor impact on the calculation results. The gap between a pair of comb fingers is designed to be 3 µm, but the fabricated result turned out to be about 3.6 µm. This will make the electrostatic force generated by the comb fingers lower than expected. The width of the spring beam is designed to be 4 µm, but the beam ended up with a width of 3.5 µm. This will reduce the stiffness of the gripper.

**Table 2:** Relationship between the displacements and voltages.

**Voltage (V)**		5.0	10.0	15.0	20.0	25.0	30.0	33.0
**Displacement (µm)**	**Simulation results**	1.2	5.0	12.0	21.0	32.5	46.8	50.0
	**Measured results**	1.0	4.0	10.0	18.0	27.0	40.0	50.0
	**Errors**	0.2	1.0	2.0	3.0	5.5	6.8	0.0

**Figure 15:**
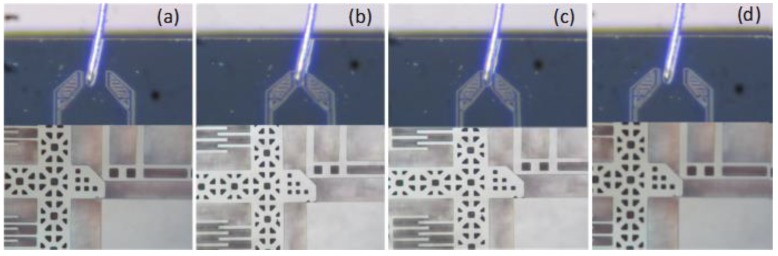
Experimental demonstration of gripping.

The performance of the designed microgripper is assessed by gripping a metal wire with a diameter of 1.6 mil (40.6 μm). The experiment is shown in Figure 15: (a) positioning the microgripper and preparing to handle the metal wire; (b) applying a driving voltage of 26.5 V to the rotary actuator to grip the metal wire; (c) continuing to hold the metal wire when the driving signal was abrogated; (d) releasing the metal wire with a voltage of 61 V on the straight comb actuators.

## 6. Conclusions

An electrostatic microgripper with a post-assembly self-locking mechanism is presented in this paper. The self-locking function of the gripper is demonstrated by gripping a metal wire with a diameter about 40.6 µm. In the experiment, the gripper successfully holds the targets without continuously applying the external driving signal, which avoids the significant effects and damage caused by driving voltage.
