# Correction: Yuan, G., *et al.* A Microgripper with a Post-Assembly Self-Locking Mechanism. *Sensors* 2015, *15*, 20140-20151

**DOI:** 10.3390/s16010080

**Published:** 2016-01-08

**Authors:** Guangmin Yuan, Weizheng Yuan, Yongcun Hao, Xiaoying Li, Honglong Chang

**Affiliations:** Key Laboratory of Micro/Nano Systems for Aerospace, Ministry of Education, Northwestern Polytechnical University, Xi’an 710072, China; yuangm@nwpu.edu.cn (G.Y.); yuanwz@nwpu.edu.cn (W.Y.); haoyongcun@126.com (Y.H.); xiaoy@nwpu.edu.cn (X.L.)

The authors wish to make the following correction to this paper [1]:

The author name “Xiaoyi Li” should be changed into “Xiaoying Li”.

The authors would like to apologize for any inconvenience caused to the readers by this change.
